# Effectiveness of Autologous Platelet Concentrates in Management of Young Immature Necrotic Permanent Teeth—A Systematic Review and Meta-Analysis

**DOI:** 10.3390/cells9102241

**Published:** 2020-10-07

**Authors:** Saurav Panda, Lora Mishra, Heber Isac Arbildo-Vega, Barbara Lapinska, Monika Lukomska-Szymanska, Shahnawaz Khijmatgar, Abhishek Parolia, Cristina Bucchi, Massimo Del Fabbro

**Affiliations:** 1 Department of Periodontics and Oral Implantology, Siksha ‘O’ Anusandhan University, Bhubaneswar 751003, India; sourav.panda@unimi.it; 2 Department of Biomedical, Surgical and Dental Sciences, Università degli Studi di Milano, 20122 Milano, Italy; khijmatgar@gmail.com; 3 Department of Conservative Dentistry and Endodontics, Institute of Dental Sciences, Siksha ‘O’ Anusandhan University, Bhubaneswar 751003, India; loramishra@soa.ac.in; 4 Department of General Dentistry, Dentistry School, Universidad San Martín de Porres, Chiclayo 14012, Peru; harbildov@usmp.pe; 5 Department of General Dentistry, Dentistry School, Universidad Particular de Chiclayo, Chiclayo 14012, Peru; 6 Department of General Dentistry, Medical University of Lodz, 92-213 Lodz, Poland; barbara.lapinska@umed.lodz.pl (B.L.); monika.lukomska-szymanska@umed.lodz.pl (M.L.-S.); 7 Nitte (Deemed to be University), AB Shetty Memorial Institute of Dental Sciences (ABSMIDS), Department of Oral Biology and Genomic Studies, Mangalore 575018, India; 8 Division of Clinical Dentistry, School of Dentistry, International Medical University, Kuala Lampur 57000, Malaysia; abhishek_parolia@imu.edu.my; 9 Faculty of Dentistry, CICO Research Centre, Universidad de La Frontera, Temuco 4811230, Chile; cristina.bucchi@ufrontera.cl; 10 Dental Clinic, IRCCS Istituto Ortopedico Galeazzi, 20161 Milano, Italy

**Keywords:** autologous platelet concentrate, meta-analysis, review, immature teeth, necrotic teeth, permanent teeth

## Abstract

The use of autologous platelet concentrates (APCs) in regenerative endodontic procedures is inconsistent and unclear. The aim of this meta-analysis was to evaluate the effectiveness of autologous platelet concentrates compared to traditional blood-clot regeneration for the management of young, immature, necrotic, permanent teeth. The digital databases MEDLINE, SCOPUS, CENTRAL, Web of Science, and EMBASE were searched to identify ten randomized clinical trials. The outcomes at postoperative follow-up, such as dentinal wall thickness (DWT), increase in root length (RL), calcific barrier formation (CB), apical closure (AC), vitality response (VR), and success rate (SR), were subjected to both qualitative synthesis and quantitative meta-analysis. The meta-analysis showed that APCs significantly improved apical closure (risk ratio (RR) = 1.17; 95% CI: 1.01, 1.37; *p* = 0.04) and response to vitality pulp tests (RR = 1.61; 95% CI: 1.03, 2.52; *p* = 0.04), whereas no significant effect was observed on root lengthening, dentin wall thickness, or success rate of immature, necrotic teeth treated with regenerative endodontics. APCs could be beneficial when treating young, immature, necrotic, permanent teeth regarding better apical closure and improved response to vitality tests.

## 1. Introduction

Until recently, the most common treatment option for immature, permanent teeth diagnosed with necrotic pulp was apexification using calcium hydroxide (CH) or mineral trioxide aggregate (MTA). CH used as a root-canal dressing, aside from involving time-absorbing treatment, was found to increase the risk of root fracture [1], whereas apical MTA plugs seem to be more effective, although expensive and difficult to handle [2]. An alternative, calcium silicate-based material, Biodentine, showed promising results, however, its immediate bonding to composite resin restoration may be impaired [3]. Unfortunately, apexification independent of the material used to produce the apical barrier does not allow for the revitalization and further root development of the immature, necrotic tooth [4], thereby compromising its prognosis [5,6].

Regenerative endodontics is an exciting new treatment modality for necrotic teeth with open apex. Murray et al. [7] described regenerative endodontic procedures (REPs) as “biologically based procedures designed to replace damaged structures” such as root and dentin, along with cells of the pulp-dentin complex. REPs aim to deliver a suitable environment (biomimetic microenvironment) in the root canal to promote repopulation of the canal with mesenchymal stem cells, such as osteo/odontoprogenitor stem cells, regeneration of pulp tissue, and continued root development [8]. Current REPs remain unable to reinstate physiological structure and function, but they can induce the development of new vascularized tissue in the root-canal space. This guided endodontic repair process allows for continuing root development, thickening of root canal walls, apical closure, and complete resolution of apical periodontitis [9].

The American Association of Endodontists proposed a regenerative endodontic protocol for the treatment of teeth with necrotic pulp and an immature apex that involves flooding the root canals with blood by overinstrumentation [10]. An alternative to creating a blood clot is the use of platelet-rich plasma (PRP), platelet-rich fibrin (PRF), or other autologous platelet concentrates (APCs).

Autologous platelet concentrates are blood derivatives (from the patient’s own blood) containing activated platelets entangled within a fibrin matrix scaffold. APCs release growth factors and cytokines that play crucial roles in the tissue regeneration process, including cell proliferation and differentiation, extracellular matrix synthesis, chemotaxis, and angiogenesis. Since these processes were found to promote healing of soft and hard tissues, APCs have been successfully used in the medical and dental fields over the last decades [11].

The classification of APCs proposed by the Periodontology, Oral Surgery, Esthetic and Implant Dentistry Organization (POSEIDO) [12] involves pure platelet-rich plasma (P-PRP), leukocyte- and platelet-rich plasma (L-PRP), pure platelet-rich fibrin (P-PRF) or leukocyte-poor, platelet-rich fibrin, and leukocyte- and platelet-rich fibrin (L-PRF).

PRP and PRF form a three-dimensional fibrin matrix and are used as REP scaffolds [13]. A blood clot contains 95% red blood cells (RBCs), 5% platelets, and <1% white blood cells (WBCs), whereas autologous platelet concentrates (PRP and PRF) contain higher concentration of platelets, which incorporate important growth factors, e.g., platelet-derived growth factor (PDGF), transforming growth factor-b (TGF-b), insulin-like growth factors (IGFs), vascular endothelial growth factor (VEGF), epidermal growth factor (EGF), and epithelial cell growth factor (ECGF), within their granules. Thus, both PRP and PRF may be a good supplement for cell-based pulp/dentin regeneration [14].

However, scientific data regarding the potential benefits of using APCs in regenerative therapies remains inconsistent. Some studies stated that both PRP and PRF positively affect only early healing of hard and soft tissues, with and without significant beneficial effect on final therapeutic outcomes [15,16,17,18,19,20,21,22]. It was also reported that the use of platelet concentrates may positively affect postoperative inflammation and pain reduction [23,24]. However, no systematic acceleration of osseous healing at the postextraction site was demonstrated, suggesting that platelet concentrates produce a negligible effect on bone regeneration [23]. True regeneration of necrotic pulp may not be able to be achieved with current techniques using PRP-type platelet concentrates, which stimulate tissue repair. In a literature review of animal studies, Del Fabbro et al. [25] concluded that there were no major or clear benefits of platelet concentrate adjuncts for pulp tissue regeneration in preclinical studies.

REPs, alongside the use of APCs, are exhibiting positive outcomes in the treatment of permanent teeth with root development. A previous systematic review of clinical studies by Lolato et al. [26] included three clinical trials and one case series to prove the beneficial effects of using APCs for the revitalization of immature, necrotic teeth. Murray [9] concluded that both PRP and PRF induce apical closure more frequently than blood-clot revascularization; however, the studies included in this review were conducted on either vital or nonvital teeth, with no forest plots or risk of bias assessments carried out to consider the study as a meta-analysis. A recent systematic review of human studies by Metlerska et al. [27] included 5 clinical trials and 21 case reports, concluding that APCs were successful in treating permanent teeth with root development. To our knowledge, no meta-analysis exists justifying the effectiveness of APCs in the management of young, immature, necrotic, permanent teeth. In the last few years, numerous clinical trials were published regarding the use of APCs in REPs, which may shed some light on their effectiveness.

Therefore, the aim of this meta-analysis was to evaluate the effectiveness of autologous platelet concentrates compared to traditional blood-clot regeneration for management of young, immature, necrotic, permanent teeth.

## 2. Materials and Methods

This review was carried out following the Preferred Reporting Items for Systematic Reviews and Meta-Analyses (PRISMA) statement guidelines [28]. The protocol of the review was registered at PROSPERO (International prospective register of systematic reviews)**,** bearing registration number CRD42020175847.

### 2.1. Search Strategy

An electronic search was carried out in five digital databases (MEDLINE, SCOPUS, CENTRAL, Web of Science, and EMBASE) using the keywords related to the topic search and combining the keywords using “AND” and “OR”. The search strategy employed was as follows: (((((((((((Autologous Platelet Concentrate) OR Platelet Rich Fibrin) OR PRF) OR Platelet rich plasma) OR PRP) OR Plasma rich in growth factors) OR PRGF) OR Concentrated growth factors) OR CGF)) AND ((((((((Revitalization) OR Revascularization) OR endodontic regeneration) OR root closure) OR open apex) OR necrotic teeth) OR young immature permanent teeth).

The search was extended to manual screening of issues in related international peer-reviewed journals, namely, International Endodontic Journal, British Dental Journal, Journal of Endodontics, Oral Surgery Oral Medicine Oral Pathology Oral Radiology and Endodontology. The bibliographies of potentially eligible clinical trials, case reports, case studies, and systematic reviews were also screened for any additional studies which were possibly fit for inclusion.

### 2.2. Study Selection

The potentially eligible articles were screened based on the inclusion criteria:

- Randomized clinical trials (RCTs);
- Studies with presence of at least one experimental group where APCs were used for treatment of young immature necrotic permanent teeth, compared to a control group using either blood-clot regeneration (BC) or any biomimetic agents;
- Studies with at least three subjects per group;
- Studies in English language only.

Exclusion criteria:

- Case reports, comments, conference proceedings, and nonrandomized clinical studies;
- Studies comparing APCs in either group with different preparation protocols or mode of delivery;
- Studies experimenting on vital teeth;
- Animal studies.

The studies retrieved from the electronic database searches and manual search were compiled into citation manager software (EndNote v7.0, Clarivate Analytics, New York, NY, USA) to remove the duplicates. After removal of duplicate items, all the studies were screened based on titles and abstracts by two independent reviewers (H.A.-V., S.P.). The potential eligible studies were subjected to full text assessment and tagged under included studies if found to satisfy the selection criteria. In cases of disagreement between the two reviewers, a third reviewer (M.D.F.) was consulted. Detailed reasons were stated for all excluded studies.

### 2.3. Data Extraction

The relevant data of the included trials were extracted in detail using an Excel spreadsheet (Microsoft, Redmond, WA, USA) independently by two review authors (A.P., C.B.) and recorded in spreadsheets. In case of missing or unclear information, the authors of the included reports were contacted by email to provide clarification regarding data given or any missing information. The following data were recorded for each of the included trials: demographic characteristics, study design and sample size, follow-up time; type of platelet concentrate used and their preparation protocol, type of intracanal medicaments and irrigation used, and study outcomes, such as dentinal wall thickness (DWT), increase in root length (RL), calcific barrier formation (CB), apical closure (AC), vitality response (VR), and success rate (SR).

### 2.4. Assessment of the Risk of Bias of the Studies

Risk of bias was assessed by two independent reviewers (L.M., S.K.) for all the included clinical trials, and discrepancies were resolved by discussion and in consultation with a third reviewer (M.L.-S.). The domains for risk assessment were graded as high, uncertain, or low risk, based on selection bias (random sequence generation and allocation concealment), performance bias (blinding), detection bias (assessor blinding), attrition bias (incomplete outcome data), and reporting bias (selective reporting). Subsequently, the overall risk for individual studies was assessed as low, moderate, or high risk based on the following criteria. The study was assessed to have a low overall risk only if all domains were found to have low risk, and high overall risk if one or more of the six domains were found to be at high risk. A moderate risk assessment was provided to the studies when one or more domains were found to be uncertain, with none at high risk.

### 2.5. Analysis of Results

The review was subjected to both qualitative and quantitative analysis based on the parameters provided in the study. The data from different studies were combined by meta-analysis only when at least two studies with similar comparisons were found, reporting the same outcome measurements at comparable observation times after intervention. The data from each study were placed and analyzed in the review manager. The statistical analysis unit was considered to be the tooth treated. If a meta-analysis could not be performed for a given outcome, then a qualitative report of the results was provided. The risk ratio (RR) for dichotomous data and the mean and standard deviation differences for continuous data were combined using random-effect models if at least four studies were available for inclusion in the meta-analysis, otherwise, a fixed-effect model was adopted.

## 3. Results

### 3.1. Selection of Studies

**Figure 1:**
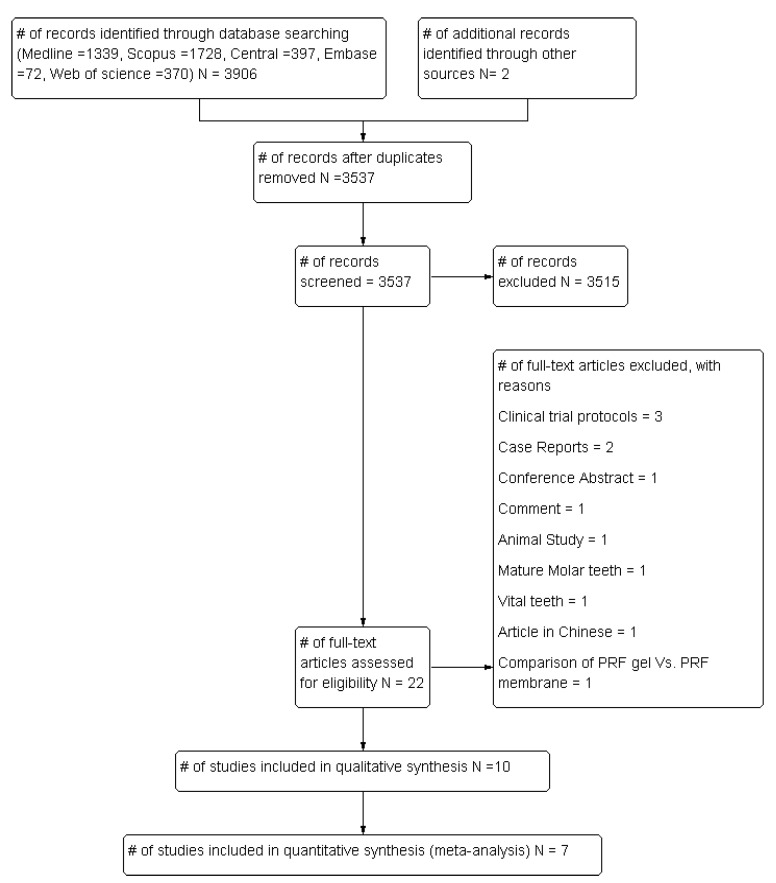
PRISMA design flowchart showing the study selection process.

This review included a total of 10 randomized clinical trials assessing the effectiveness of APCs in the management of young, immature, necrotic, permanent teeth [29,30,31,32,33,34,35,36,37,38]. The included articles were identified from a pool of 3537 articles retrieved from digital databases and a manual search, after removal of duplicates. Full text assessments were carried out for 22 articles, and 12 studies were excluded due to reasons reported in Figure 1.

### 3.2. Characteristics of the Studies

The included trials were published between 2012 and 2020. The trials included 303 participants with an age range of 7–28 years, accounting for 319 necrotic, immature, permanent teeth (particularly incisors and premolars). The etiology of pulpal necrosis was found mostly secondary to caries or trauma. Only 1 trial [29] included teeth with developmental anomalies (dens invaginatus). The minimum follow-up duration among the included trials was 12 months. Two studies [36,37] reported postoperative measurements at the end of 18 months, and another trial recorded postoperative reading “till they achieved complete healing” [32]. The follow-up duration for the latter study ranged from 10–49 months. The general characteristics of the included trials are provided in Table 1.

Some details on the operative protocols of each included study are reported in Table 2.

### 3.3. Analysis of Risk of Bias of the Studies

Only two studies [29,34] were assessed to be at low risk, whereas eight studies [30,31,32,33,35,36,37,38] were at moderate risk of bias (Table 3).

There was uncertainty in defining proper allocation concealment in most of the studies [30,32,35,36,37,38], along with blinding in few studies [31,32,33,35,36,37,38]. The aforementioned reasons led to moderate overall risk assessment in the above cited studies.

### 3.4. Synthesis of Results (Meta-Analysis)

The meta-analysis was carried out with quantitative outcome data extracted from seven included trials [30,31,32,34,35,36,38]. A blobbogram of the respective outcomes was plotted to compare the effectiveness of APCs in comparison to BC for treatment of young, immature, necrotic, permanent teeth.

The meta-analysis was carried out in review manager software (RevMan, version 5.3; Nordic Cochrane Centre (Cochrane Collaboration), Copenhagen, Denmark; 2014). Forest plots were attempted for dentinal wall thickness (DWT), increase in root length (RL), calcific barrier formation (CB), apical closure (AC), vitality response (VR), and success rate (SR)., and sub-group analysis was carried out based on the type of platelet concentrate used (PRP/PRF).

#### 3.4.1. Dentinal Wall Thickness (DWT)

**Figure 2:**
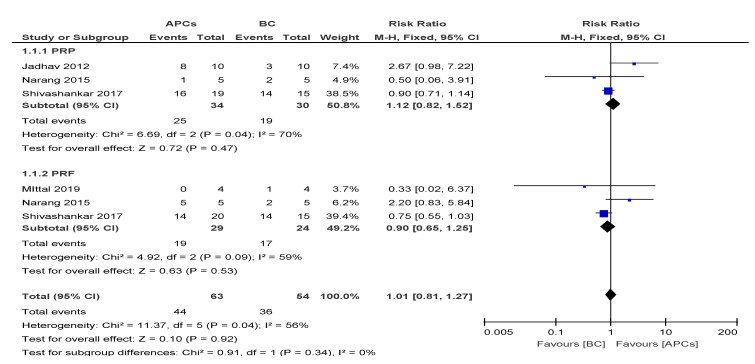
Forest plot showing a comparison of the dentinal wall thickness.

The overall risk ratio (RR = 1.01, 95% CI: 0.81, 1.27) of achieving excellent/good DWT was found to be not significant (*p* = 0.92) between the use of APCs and BC in the management of young, immature, necrotic, permanent teeth (Figure 2).

#### 3.4.2. Increase in Root Length (RL)

**Figure 3:**
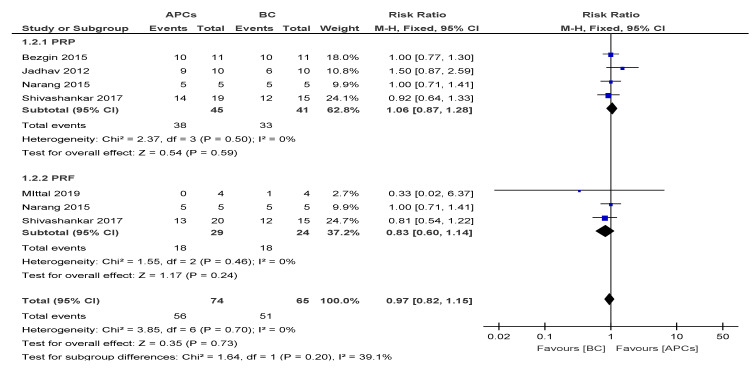
Forest plot showing a comparison of the increase in root length (RL).

The overall risk ratio (RR = 0.97, 95% CI: 0.82, 1.15) of achieving excellent/good RL was found to be not significant (*p* = 0.73) between the use of APCs and BC in the management of young, immature, necrotic, permanent teeth (Figure 3).

#### 3.4.3. Apical Closure (AC)

**Figure 4:**
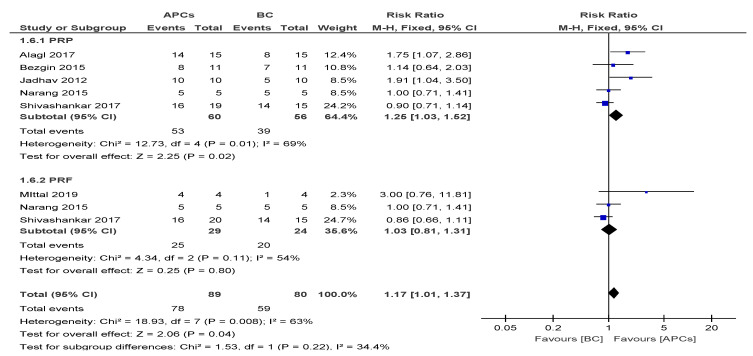
Forest plot showing a comparison of apical closure (AC).

The overall risk ratio (RR = 1.17, 95% CI: 1.01, 1.37) of achieving excellent/good AC was found to not be significant (*p* = 0.04) between the use of APCs and BC in the management of young, immature, necrotic, permanent teeth (Figure 4).

The sub-group analysis suggested a better response of the PRP group (RR = 1.25; 95% CI: 1.03, 1.52; *p* = 0.01) in achieving AC than PRF group.

#### 3.4.4. Vitality Response

**Figure 5:**
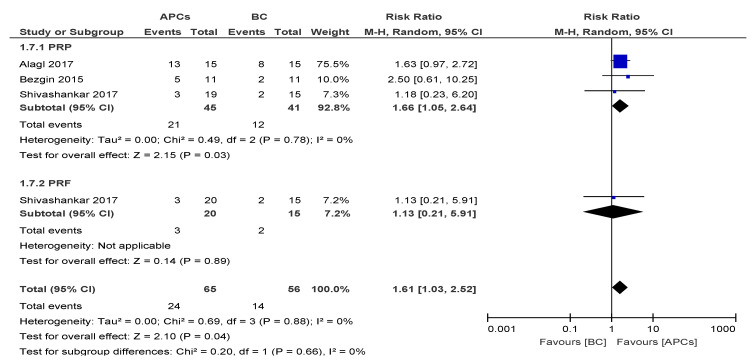
Forest plot showing a comparison of the vitality response (VR).

The overall risk ratio (RR = 1.61; 95% CI: 1.03, 2.52) of achieving excellent/good AC was found to be significant (*p* = 0.04) between the use of APCs and BC in the management of young, immature, necrotic, permanent teeth (Figure 5).

The sub-group analysis suggested a better response of the PRP group (RR = 1.66; 95% CI: 1.05, 2.64; *p* = 0.03) in achieving AC than the PRF group.

#### 3.4.5. Success Rate (SR)

**Figure 6:**
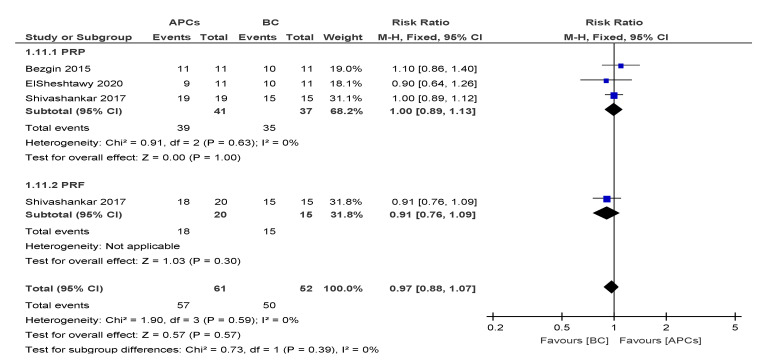
Forest plot showing a comparison of success rates (SR).

The overall risk ratio (RR = 0.97; 95% CI: 0.88, 1.07) of achieving excellent/good SR was found to be not significant (*p* = 0.57) between the use of APCs and BC in the management of young, immature, necrotic, permanent teeth (Figure 6).

#### 3.4.6. Calcific Barrier Formation (CB)

Only one study [31] recorded calcific barrier formation and found that 8 out of 11 teeth treated with PRF showed calcific barrier formation, among which seven teeth developed an apical barrier and only one tooth developed a cervical barrier at the end of 12 months of follow-up. Similar data were reported for the group treated with BC. However, in this group, five teeth showed apical barrier formation and three teeth showed cervical barrier formation.

## 4. Discussion

Revitalization, also called regenerative endodontics, is a promising therapy for immature necrotic teeth, with excellent clinical outcomes and a success rate of over 90%, according to a recent, large clinical trial [40]. While most treated teeth showed root maturation, healing of periapical lesions, and resolution of patient symptomatology [4,26,40], nearly half also showed positive responses to pulp vitality tests [40], indicating that the newly developed/formed tissue was vascularized and could therefore support sensory innervation. This systematic review aimed to evaluate whether the use of autologous platelet concentrates in the root canal of immature necrotic teeth improved the clinical outcomes of revitalization.

In general terms, the clinical protocol for revitalization indicates debridement and disinfection of the tooth with use of sodium hypochlorite in low concentrations (2–2.5%) during the first appointment, with minimally invasive or no instrumentation, followed by intracanal medication with calcium hydroxide or triple antibiotic paste (TAP). During the second appointment, the canal should be irrigated with EDTA and bleeding should be evoked to recruit stem cells from periapical tissues and provide an scaffold for the ingrowth of new tissue [41]. Although this is a very simple and straightforward approach which eases the widespread use of the therapy, it may also carry some drawbacks and limitations. The formation of a blood clot in the canal is not always predictable, and/or the blood may contaminate the crown of the tooth and cause discoloration [42]. Additionally, limitations in the disinfecting agents and the nature of dentin cause bacteria to remain present in the dentin after debridement [43]. Consequently, most failed cases after revitalization are due to remnant infection [44]. In this context, autologous platelet concentrates are an interesting and cost-effective method for regenerative endodontics due to their high concentration of growth factors that induce migration, proliferation, and differentiation of stem cells, their dense fibrin matrix that serves as a stable scaffold, and their bacteriostatic properties [45].

This systematic review confirmed that regenerative endodontics is a reliable option for the treatment of immature necrotic teeth, as the overall success rate was 96.5%. However, it must be noticed that the follow-up periods of the included studies were short. All but one study [32] included in this systematic review had a follow-up period of 12 or 18 months. Clinical trials evaluating the success rate of regenerative endodontics with a follow-up period of five years or more are currently lacking in the literature.

The meta-analysis conducted in this systematic review concluded that APCs significantly improved apical closure and response to vitality pulp tests, whereas no significant effects were observed on root lengthening, dentin wall thickness, or the success rate of immature, necrotic teeth treated with regenerative endodontics. The positive effects of APCs regarding their response to the vitality pulp test were also found in a previous systematic review [27]. In the subgroup analysis, teeth treated with PRP showed better apical closure and response to the vitality pulp test compared to those treated with PRF. A possible hypothesis for PRF not always being as effective as PRP could be due to the different bioactivities of the two APCs. PRF is composed of a dense and stable fibrin network [46] that allows slower release of growth factors compared to PRP. Thus, PRP releases significantly more growth factors when compared to PRF during the first 15–60 min after clot formation, while PRF displays a continual and steady release of a modest amount of growth factors over a 10-day period [47]. Such high concentrations of bioactive molecules released by PRP could be responsible for the apparent beneficial effects over PRF, at least in the short-term. Only one study [34] assessed tooth response to the vitality test when treated with both PRP and PRF, showing a comparable response. No other study investigated the effect of PRF on this variable. From these observations, it could be concluded that there is a trend of PRP showing better results than PRF in regenerative endodontic procedures. However, more clinical studies with large sample sizes are urgently needed to confirm or deny this trend over a long follow-up time period.

One of the first considerations emerging from this review is the moderate risk of bias present in most of the included studies. Most of the studies failed to ensure concealment of allocation, and three failed to ensure blinding of the outcome assessment [35,36,38]. Moreover, due to the nature of the treatment, most studies found it impossible to ensure blinding of the patient and personnel, because the patients receiving platelet concentrates knew which group they were assigned to, since they were submitted to blood drawing. This bias could be resolved by conducting split mouth design studies where possible. Although it is not possible to determine if and how such a lack of blinding affected the outcomes, further clinical trials on regenerative endodontics should consider this, since flaws in the design of clinical trials may bias intervention effect estimates and increase between-trial heterogeneity [47].

The included clinical trials presented important differences in protocols regarding treatment performance. Most studies did not follow the standardized protocol for revitalization, as published by the American Association of Endodontists [8] and the European Society of Endodontics [41]. Among the variations, with respect to the standard treatment, was the use of sodium hypochlorite in high concentrations (5.25%) [29,31,34], the lack of use of EDTA [30,31,34,36,37], and the use of triple antibiotic pastes with high concentrations and/or those containing minocycline [33,34,35]. All these variations may have affected the clinical outcomes of revitalization. High concentrations of sodium hypochlorite reduce the viability of stem cells and their odontogenic/osteogenic differentiation [48]. EDTA, on the other hand, reduces the deleterious effect of sodium hypochlorite and improves cell survival and differentiation [48]. Moreover, EDTA liberates the growth factors present in dentin that positively affect stem cell adhesion, migration, and differentiation [49]. TAP, especially in high concentrations, has cytotoxic effects on stem cells and reduces mineralization [50], and, when minocycline is included, can cause significant tooth discoloration [51]. Heterogeneity was also present in the protocol to obtain the autologous platelet concentrates. Studies used different rotations per minute (RPM), activators and anticoagulants, and numbers of centrifugation steps to obtain the concentrates. Further studies should use the standardized published protocol for REPs together with standardized protocols for APCs, which would facilitate the comparison of results among the trials.

## 5. Conclusions

APCs significantly improved apical closure and response to vitality pulp tests, whereas no significant effects were observed on root lengthening, dentin wall thickness, or success rate of immature, necrotic teeth treated with regenerative endodontics. Further studies with standardized protocols are necessary to assess the actual contribution of APCs in regenerative endodontics.

## Figures and Tables

**Table 1:** General Characteristics of the included studies.

Author	Type of Study	Country	*n* patient	*n* Teeth	Groups of Study	*n* per Group	Tooth	Etiology of Pulpal Necrosis	Follow-up Time in Months
ElSheshtawy et al. 2020 [29]	RCT, Parallel	Egypt	26	26	A. PRP	11	Incisor	Secondary to trauma and dens invaginatus	12
					B. BC	11			
Mittal et al. 2019 [30]	RCT, Parallel	India	16	16	A. PRF	4	Incisor	Secondary to trauma/caries	12
					B. BC	4			
Ragab et al. 2019 [31]	RCT, Parallel	Egypt	22	22	A. PRF	11	Incisor	Secondary to trauma	12
					B. BC	11			
Ulusoy et al. 2019 [32]	RCT, Parallel	Turkey	77	77	A. PRP	18	Incisor	Secondary to trauma	Until complete healing 10–49
					B. PRF	17			
					C. PP	17			
					D. BC	21			
Rizk et al. 2019 [33]	RCT, Parallel	Egypt	26	26	A. PRP	13	Incisor	Secondary to trauma	12
					B. PRF	12			
Shivashankar et al. 2017 [34]	RCT, Parallel	India	60	60	A. PRF	20	Incisor	Secondary to trauma/caries	12
					B. BC	15			
					C. PRP	19			
Alagl et al. 2017 [35]	RCT, Split Mouth	Saudi Arabia	16	32	A. PRP	15	Incisor and premolars	Secondary to trauma/caries	12
					B. BC	15			
Bezgin et al. 2015 [36]	RCT, Parallel	Turkey	20	22	A. PRP	11	Incisor and premolars	Secondary to trauma/caries	18
					B. BC	11			
Narang et al. 2015 [37]	RCT, Parallel	India	20	20	A. MTA	5	NR	Secondary to trauma/caries	18
					B. BC	5			
					C. PRF	5			
					D. PRP	5			
Jadhav et al. 2012 [38]	RCT, Parallel	India	20	20	A. PRP	10	Incisor	Secondary to trauma/caries	12
					B. BC	10			

Legend: RCT: randomized clinical trial; PRP: platelet-rich plasma; BC: induced blot clot; PRF: platelet-rich fibrin; PP: platelet pellet; MTA: mineral trioxide aggregate.

**Table 2:** Details on the operative protocol of the included studies.

Author	Presence of Periapical Lesion	Instrumentation	Irrigation Methods	Intra-Canal Medication	Recall Time (Weeks)	Preparation Protocol of APC	Access Restoration
ElSheshtawy et al. 2020 [29]	Yes	No	20 mL of 5.25% sodium hypochlorite. At recall, 20 mL of 2.5% sodium hypochlorite, followed by 20 mL sterile saline and 10 mL of 17% EDTA solution.	Triple Antibiotic Paste	NR	PRP was prepared according to Dohan et al. [39], after which concentrated platelet-rich plasma (cPRP) was prepared and introduced inside dry root canals using a sterile 30 G syringe. The canal was then backfilled with cPRP to a level just beneath the CEJ and left to clot for 10 min.	MTA, using a layer of reinforced glass ionomer (Riva self-cure, SDI limited, Bayswater, Victoria, Australia) followed by resin composite (Filtek z250 universal restorative, 3 mol L 1ESPE, St. Paul, MN, USA).
Mittal et al. 2019 [30]	Yes	Minimal (#30 K file)	2.5% sodium hypochlorite (copious irrigation).	Double Antibiotic Paste	4 weeks	PRF was prepared by drawing 5 mL of venous blood from the patient, collected in a dried glass test tube, and centrifuged at 2700 rpm for 12 min.	Glass ionomer cement followed by composite resin.
Ragab et al. 2019 [31]	Yes	No	20 mL of 5.25% sodium hypochlorite followed by 20 mL sterile saline.	Double Antibiotic Paste	3 weeks	PRF was prepared by drawing 12 mL sample of whole blood intravenously from the patient’s right antecubital vein and centrifuged under 3000 rpm for 12 min.	MTA plus Light Cure Glass ionomer cement.
Ulusoy et al. 2019 [32]	Yes	No	20 mL 1.25% sodium hypochlorite. At recall, 2% chlorhexidine, saline and 1 mL 17% EDTA.	Triple Antibiotic Paste	4 weeks	PRP: Citrated blood was centrifuged in a standard laboratory centrifuge PK 130 (ALC International; Cologno Monzese, Italy) for 15 min at 1250 revolutions per minute (rpm) to obtain PRP without erythrocytes and leukocytes. PRF: 10 mL blood was collected in a sterile tube without anticoagulant and centrifuged immediately for 10 min at a speed of 3000 rpm (Andreas Hettich Group, Ltd., Tuttlingen, Germany).	MTA coronal barrier was sealed with a thin glass ionomer base, and final coronal restorations were placed at the same visit using acid etch composite resin.
Rizk et al. 2019 [33]	Yes	No	20 mL 2% sodium hypochlorite for 5 min, followed by 20 mL 17% EDTA.	Triple Antibiotic Paste	3 weeks	PRP was prepared according to the description by Dohan et al. [39]. PRP was combined with equal volumes of sterile solution containing 10% calcium chloride and sterile bovine thrombin (100 U/mL) to achieve coagulation. PRF: 10 mL blood was collected in a sterile tube without anticoagulant and centrifuged immediately for 10 min at a speed of 3000 rpm.	An MTA orifice plug extending 2–3 mm in the canal was used to seal the canal orifice then glass ionomer (GC America, Alsip, IL) and composite (Z 250, 3 M ESPE) to give an effective and durable seal.
Shivashankar et al. 2017 [34]	No	Minimal	5.25% sodium hypochlorite (copious irrigation).	Triple Antibiotic Paste	3 weeks	NR	NR
Alagl et al. 2017 [35]	Yes	No	2.5% sodium hypochlorite (20 mL), sterile saline (20 mL), and 0.12% chlorhexidine (10 mL), followed by 17% EDTA after 3 weeks.	Triple Antibiotic Paste	3 weeks	PRP was prepared according to the description by Dohan et al. [39]. PRP was combined with equal volumes of sterile solution containing 10% calcium chloride and sterile bovine thrombin (100 U/mL) to achieve coagulation.	NR
Bezgin et al. 2015 [36]	Yes	No	2.5% sodium hypochlorite (20 mL), sterile saline (20 mL), and 0.12% chlorhexidine (10 mL), followed by 5% EDTA (20 mL) after 3 weeks.	Triple Antibiotic Paste	3 weeks	PRP was prepared according to the description by Dohan et al. [39]. PRP was combined with equal volumes of sterile solution containing 10% calcium chloride and sterile bovine thrombin (100 U/mL) to achieve coagulation.	Final restoration was completed with white MTA (Angelus, Londrina, Brazil), reinforced glass ionomer cement (Ketac Molar Easymix; 3M ESPE, Seefeld, Germany), and composite resin (Filtek Supreme XT; 3M ESPE, St Paul, MN, USA).
Narang et al. 2015 [37]	Yes	Minimal	2.5% sodium hypochlorite (copious irrigation)	Triple Antibiotic Paste	4 weeks	NR	Resin-modified glass ionomer cement was placed extending 3–4 mm in the canal. Access cavity was sealed with composite (Clearfil Majesty, Kuraray Medical Inc., Tokyo, Japan).
Jadhav et al. 2012 [38]	No	Minimal (#60 H file)	2.5% sodium hypochlorite (copious irrigation).	Triple Antibiotic Paste	NR	PRP: 8 mL of blood drawn by venipuncture of the antecubital vein was collected in a 10 mL sterile glass tube coated with an anticoagulant (acid citrate dextrose) and centrifuged at 2400 rpm for 10 min to separate PRP and platelet-poor plasma (PPP) from the red blood cell fraction. The top-most layer (PRP + PPP) was transferred to another tube and again centrifuged at 3600 rpm for 15 min to separate the PRP to precipitate at the bottom of the glass tube. This was mixed with 1 mL 10% calcium chloride to activate the platelets and to neutralize the acidity of acid citrate dextrose.	Resin-modified glass ionomer cement (Photac-Fill; 3M ESPE, St Paul, MN, USA)

Legend: NR: not reported, EDTA: ethylene di-amine tetra-acetic acid, PRP: platelet-rich plasma, PRF: platelet-rich fibrin, CEJ: cementoenamel junction, MTA: mineral trioxide aggregate.

**Table 3:** Risk of bias assessment of included trials.

Author.	Year	Random Sequence Generation (Selection Bias)	Allocation Concealment (Selection Bias)	Blinding of Participants and Personnel (Performance Bias)	Blinding of Outcome Assessment (Detection Bias)	Incomplete Outcome Data (Attrition Bias)	Selective Reporting (Reporting Bias)	Overall Risk
ElSheshtawy et al. 2020 [29]	2020	Low	Low	Low	Low	Low	Low	Low
Mittal et al. 2019 [30]	2019	Low	Unclear	Low	Low	Low	Low	Moderate
Ragab et al. 2019 [31]	2019	Low	Unclear	Unclear	Low	Unclear	Low	Moderate
Rizk et al. 2019 [33]	2019	Low	Low	Unclear	Low	Low	Low	Moderate
] Ulusoy et al. 2019 [32]	2019	Low	Unclear	Unclear	Low	Low	Low	Moderate
Alagl et al. 2017 [35]	2017	Low	Unclear	Unclear	Unclear	Low	Low	Moderate
Shivashankar et al. 2017 [34]	2017	Low	Low	Low	Low	Low	Low	Low
Bezgin et al. 2015 [36]	2015	Low	Unclear	Unclear	Unclear	Low	Low	Moderate
Narang et al. 2015 [37]	2015	Low	Unclear	Unclear	Low	Low	Low	Moderate
Jadhav et al. 2012 [38]	2012	Low	Unclear	Unclear	Unclear	Low	Low	Moderate
